# High Failure Rates of Melarsoprol for Sleeping Sickness, Democratic Republic of Congo

**DOI:** 10.3201/eid1406.71266

**Published:** 2008-06

**Authors:** Jo Robays, Gaspard Nyamowala, Claude Sese, Victor Betu Ku Mesu Kande, Pascal Lutumba, Wim Van der Veken, Marleen Boelaert

**Affiliations:** * Institute of Tropical Medicine, Antwerp, Belgium; † Programme National de Lutte contre la Trypanosomiase Humaine Africaine, Kinshasa, Democratic Republic of Congo; ‡ Medische Missie Samenwerking Organisation, Kinshasa; § University of Kinshasa, Kinshasa; ¶ Belgian Technical Cooperation, Kinshasa

**Keywords:** Human African trypanosomiasis, relapse, treatment, melarsoprol, dispatch

## Abstract

A retrospective chart review of 4,925 human African trypanosomiasis patients treated with melarsoprol in 2001–2003 in Equateur Nord Province of the Democratic Republic of Congo showed a treatment failure rate of 19.5%. This rate increased over the 3 years. Relapse rates were highest in the central part of the province.

Human African trypanosomiasis (HAT or sleeping sickness), caused by *Trypanosoma brucei gambiense,* is a slowly progressing fatal infectious disease that affects an estimated 100,000 persons in Central Africa each year. In the meningoencephalitic or second stage, melarsoprol, an arsenic derivative, and eflornithine are the only effective drug treatments available (*1*). High relapse rates for patients treated with melarsoprol were documented in Uganda (*2**,**3*) and in M’banza Congo in Angola (*4*). We investigated recent reports about very high relapse rates from the province of North Equator, in the Democratic Republic of Congo (DRC), where the largest HAT epidemic in recent history occurred (*5*).

## The Study

We reviewed the records of all patients who received HAT treatment in the period January 1, 2001–December 31, 2003, in the 23 treatment centers operating in North Equator Province. We included only those case-patients who had received complete treatment with melarsoprol, obtained from Sanofi Aventis (Paris, France) under the World Health Organization donation program with 1 of the following regimens: 3 series of 3 injections (3.4 mg/kg) at 7-day intervals for patients with a cerebrospinal fluid (CSF) leukocyte count >20 leukocytes/mm^3^, or 2 series of 3 injections (3.4 mg/kg) at 7-day intervals for patients with CSF leukocyte counts of 5–20 leukocytes/mm^3^. Patients were asked to return for a routine follow-up visit to the HAT treatment center at 6, 12, 18, and 24 months after treatment or any time they felt unwell between visits.

Age, sex, disease stage, and results of parasitologic tests of the patients who experienced a relapse were recorded during the chart review. HAT relapse was defined as follows: trypanosomes found in body fluids at any follow-up assessment or a CSF leukocyte count >20/mm^3^ and twice as high as the count at the previous follow-up visit. This case definition for relapse is used by the national program and in clinical trials (*6*). Patients who did not show up for suggested follow-up visits were not visited at home.

The relapse rate was calculated as the number of patients with HAT who experienced a relapse, divided by all patients who received full melarsoprol treatment during the study period. The study included 4,925 patients with second-stage parasitologically confirmed HAT; all were treatment-naïve for HAT, and none had been referred by another center. Relapse after melarsoprol treatment was noted for 959 (19.5%) patients. Table 1 shows the relapse rate by geographic area for the years 2001, 2002, and 2003. The patients from the central part of the province showed the highest relapse rates, and the trend increased over the 3 years (χ^2^ for trend 22.3, p<0.001).

**Table 1:** Melarsoprol relapse rates in second-stage human African trypanosomiasis patients, Equateur Nord Province, 2001–2003

Year	Part of province									Total	
	Northern			Central			Southern				
	No. treated	No. (%) relapsed		No. treated	No. (%) relapsed		No. treated	No. (%) relapsed		No. treated	No. (%) relapsed
2001	708	90 (12.7)		1,154	284 (24.6)		506	46 (9.1)		2,368	420 (17.7)
2002	572	56 (9.8)		799	180 (22.5)		135	36 (26.7)		1,506	272 (18.1)
2003	362	57 (15.7)		570	171 (30.0)		119	39 (32.8)		1,051	267 (25.4)
Total	1,642	203 (12.4)		2,523	635 (25.2)		760	121 (15.9)		4,925	959 (19.5)

Table 2 shows characteristics of the patients with a relapse of HAT after melarsoprol treatment. Lumbar puncture was performed for 92% of patients at 6 months. This proportion dropped to 73% after 2 years, according to the annual reports for 2001–2005. Only 4.8% of patients with first-stage illness experienced a relapse; direct evidence of the parasite was found in 27% of these case-patients.

**Table 2:** Characteristics of 959 patients who experienced relapse after treatment  with melarsoprol, Equateur Nord Province, 2001–2003

Characteristic	No. (%)
Sex	
M	433 (45.2)
F	526 (54.8)
Age	
>1–11mo	1 (0.1)
12–59 mo	13 (1.4)
5–59 y	936 (97.6)
>60 y	9 (0.9)
Stage of disease	
First	46 (4.8)
Second	782 (81.5)
Unknown	131 (13.7)
Parasites found	259 (27.0)

## Conclusions

These data show that high failure rates with melarsoprol are no longer limited to Kasai Province in DRC. The HAT focus of the Equateur Nord Province does not border that in the Kasai Province, and given the limited contact between both provinces, resistant strains likely did not spread from Kasai to Equateur Nord Province.

The reported failure rate is certainly underestimated because some of the patients who did not return for follow-up visits probably died at home or sought treatment elsewhere. We cannot exclude the possibility that some retreated patients had been reinfected. However, the trend in observed incidence rates in Equateur Nord Province had been declining since 2004, and, regardless, the incidence was too low to contribute substantially to the observed 19.5% failure rate. The program used only basic parasitologic confirmation tests and none of the more sensitive concentration techniques, such as capillary tube centrifugation, quantitative buffy coat test, or the miniature anion-exchange column for trypanosomiasis during the study period, which explains the low proportion of relapses that were parasitologically confirmed.

Melarsoprol was used on a massive and unprecedented scale in Equateur Nord Province from 1996 through 2005, when 38,945 new patients received treatment with melarsoprol (annual reports of national program 2000 and 2005), so drug pressure (i.e., the use of a certain antimicrobioal agent potentially selecting out resistant strains) was certainly present in the region. Similarly high failure rates with melarsoprol in the Kasai Province led the national control program to introduce eflornithine as a first-line treatment there in 2006. The cause of these high relapse rates remains unclear because until now melarsoprol resistance could not be demonstrated in parasites in vitro or in animal models (*5*). The program used a shorter melarsoprol treatment regimen than that used by other countries, where 3 series of 4 injections are used. This difference is unlikely to be an explanation for the high relapse rates found, however, as in other provinces of DRC, relapse rates with the same regimen remained considerably lower (e.g., a 1.4% reported relapse rate for Bandundu Province, according to the 2006 Annual Report of the national control program).

Given this high failure rate, a switch to the safer eflornithine regimen is the most obvious solution. However, this drug is more complex to administer: patients receiving it require intravenous fluids and a high standard of nursing care. Eflornithine offers the additional advantage of lesser toxicity, which might enhance the acceptability of HAT treatment. A cost-effectiveness analysis showed that eflornithine is the more cost-effective option whenever relapse rates with melarsoprol treatment exceed 15% (*7*). Legitimate concerns have been raised regarding the use of this drug in monotherapy as first-line treatment because there are no alternatives if resistance to it emerges. A clinical trial on the use of the combination DFMO (alpha-difluoromethylornithine)–nifurtimox is in progress (*8*). Our data show the urgent need for novel drugs for stage-2 HAT. Public-private partnerships such as the Drugs for Neglected Diseases Initiative and others are currently investing in development of such drugs, but a new drug will not likely be available within the next decade. Therefore, rapidly controlling new outbreaks is essential to prevent large epidemics. These outbreaks may be more difficult to control if eflornithine resistance emerges.
